# Wood-Poly(furfuryl Alcohol) Prepreg: A Novel, Ecofriendly Laminate Composite

**DOI:** 10.3390/ma16186237

**Published:** 2023-09-15

**Authors:** Andrey Pereira Acosta, Bruno Esteves, Joziel Aparecido da Cruz, Arthur Behenck Aramburu, Agnė Kairytė, Sylwia Członka, Dionatan Orestes Ramos, Matheus de Paula Goularte, Rafael de Avila Delucis, Darci Alberto Gatto, Sandro Campos Amico

**Affiliations:** 1 Postgraduate Program in Mining, Metallurgical and Materials Engineering, Federal University of Rio Grande Do Sul, Porto Alegre 90040-060, RS, Brazil; andrey.acosta@ufrgs.br (A.P.A.); joziel.cruz@ufrgs.br (J.A.d.C.); arthuraramburu@gmail.com (A.B.A.); amico@ufrgs.br (S.C.A.); 2 Department of Wood Engineering, Polytechnic Institute of Viseu, Av. Cor. José Maria Vale de Andrade, 3504-510 Viseu, Portugal; bruno@estgv.ipv.pt; 3 Laboratory of Thermal Insulating Materials and Acoustics, Institute of Building Materials, Faculty of Civil Engineering, Vilnius Gediminas Technical University, Linkmenų St. 28, 08217 Vilnius, Lithuania; 4 Institute of Polymer and Dye Technology, Faculty of Chemistry, Lodz University of Technology, Stefanowskiego 12/16, 90-924 Lodz, Poland; sylwia.czlonka@dokt.p.lodz.pl; 5 Chemical Engineering, Federal University of Rio Grande Do Sul, Porto Alegre 90040-060, RS, Brazil; dionatanor@gmail.com; 6 Postgraduate Program in Materials Science and Engineering, Federal University of Pelotas, Pelotas 96010-150, RS, Brazil; almatheusgoularte@gmail.com (M.d.P.G.); rafael.delucis@ufpel.edu.br (R.d.A.D.); darci.gatto@ufpel.edu.br (D.A.G.)

**Keywords:** vegetable fibers, wood laminates, ecofriendly resin, ecofriendly composite, pine wood

## Abstract

Prepregs are commonly fabricated with non-renewable petroleum-based materials. To reduce the impact of the manufacturing of these materials and to produce more sustainable prepregs, this research aims to manufacture poly(furfuryl alcohol)/wood veneer prepregs and their posterior molding in laminate composites. For this purpose, the vacuum infusion process was used to impregnate the wood veneers, and compression molding was applied to manufacture three- and four-layer laminate composites. Scanning electronic microscopy was used to evaluate the impregnation. the laminate manufacturing and differential scanning calorimetry were used to predict the shelf-life of the prepregs, Fourier-transform infrared was used to evaluate the induced hydrolysis resistance, and thermogravimetric analysis was used to determine the thermal degradation of the laminates. Moreover, water uptake and flexural, compressive, and tensile properties were evaluated. The kinetic models were effective and showed a shelf life for the laminates of approximately 30 days in storage at −7 °C, which is an interesting result for laminates with lignocellulosic materials. FTIR proved the laminates’ excellent resistance to hydrolysis. The water absorption, thermal stability, and mechanical properties did not differ as the amount of wood veneer increased, but these results were up to ~40% higher compared with unidirectional wood laminates found in the literature, which is probably linked to the excellent interface observed with SEM.

## 1. Introduction

Prepregs are composite materials composed of reinforcing fibers, such as carbon, glass, or aramid fibers, that have undergone pre-impregnation with a partially cured resin. Subsequently, these materials are meticulously stacked in a prescribed sequence and subjected to the curing process, resulting in the formation of a definitive laminate composite structure [1]. Prepregs present a distinct advantage in their ability to achieve precise control over the resin-to-fiber ratio, facilitating the attainment of high-fiber-volume fractions. This attribute contributes to enhanced strength and stiffness characteristics. Furthermore, prepregs exhibit superior handling and processing properties, rendering them well suited for a diverse range of applications across industries, including the aerospace, automotive, sports equipment, and marine sectors [2,3,4].

While these materials provide exceptional performance in composites, they are not commonly sustainable materials. In fact, the most commonly used resin systems for this application (e.g., epoxy resin) have manufacturing processes involving the use of non-renewable petrochemicals and require high energy consumption [4]. Thereafter, the synthetic fibers used as reinforcement also involve a complex process that requires high temperatures and large amounts of energy, which contribute to significant greenhouse gas emissions [5]. Thus, in recent years, there has been an increasing interest in the development of prepregs that incorporate renewable and biodegradable materials. In this sense, sustainable materials, such as wood veneers [6,7,8,9] or natural fibers, as potential reinforcement materials in prepregs are being explored [10,11,12], offering benefits such as reduced environmental impact, increased biodegradability, and renewability.

Natural fibers, including flax [13], curauá [12], and kenaf [14], have been developed as prepreg reinforcement materials with significant progress in various applications. In this sense, Silva et al. [10] manufactured prepreg composites using sisal fibers and epoxy resin, wherein the addition of sisal fibers to the composite resulted in a significant increase in tensile and flexural strength, concluding that the prepreg manufacturing process is a viable and effective alternative for the production of lignocellulosic, fiber-reinforced composites. Moreover, wood veneers are promising materials for prepregs given their excellent mechanical properties, high stiffness, low weight, low carbon footprint, and renewability. In fact, Mohammadabadi et al. [6] employed vacuum-assisted resin transfer molding (VARTM) in combination with low-viscosity thermoplastic resin to fabricate wood-based prepregs. Their findings demonstrate encouraging outcomes, suggesting that this approach serves as a viable alternative to conventional wood-composite-forming techniques. Furthermore, this method exhibits the potential to streamline the production of intricate geometries while also enhancing the inherent properties of the natural material.

However, the use of natural fibers may complicate the production of composite panel products with complex geometries for high-end applications, such as in the automotive and aerospace industries [6]. Conversely, wood veneers have an advantage over other natural fibers and have great potential for industrial proposes given their eco-friendliness and lightweight composite properties. In fact, Mohammadabad et al. [6] studied thermoplastic wood-based prepreg using the VARTM process and stated that low-viscosity resins may result in high-performance thin-wood prepregs. In this sense, the main disadvantage of wood veneers, as well as natural fibers, is their low permeability compared with traditional materials for this purpose, e.g., the permeability of wood veneers is 100 times lower than glass fibers [15,16,17].

Moreover, although good results have been reported, another limitation of composites with wood fibers and veneers is downsides such as hydrolysis, which impairs adhesion with the matrix [18]. In addition, the use of non-renewable resins for pre-impregnated materials is very common. In this sense, furfuryl alcohol (FA) is a thermosetting polymer derived from furfural, which is obtained from renewable sources such as lignocellulosic biomass, making it a highly sustainable alternative to petroleum-derived polymers [19]. Furthermore, FA has good thermal stability, low density, and high mechanical strength, which makes it a viable alternative for producing prepregs [20]. For this purpose, the study of the curing kinetics of resins is important for the development and optimization of the cure cycles used in the manufacturing process of composite materials. Curing kinetic models can be used to simulate the curing process, and in this way, the cycle time can be minimized [21].

Thus, the use of sustainable materials in prepregs has shown great potential in creating ecofriendly composite materials. The development of prepregs using wood veneers has the potential to offer a sustainable and environmentally friendly alternative to traditional materials. Moreover, the use of renewable resins synergistically with wood veneers for the manufacture of pre-impregnated materials for the subsequent production of ecofriendly polymeric composites is an interesting alternative since it is possible to manufacture the shape of the desired structural part in loco. Thus, this research examines the first stage of manufacturing poly(furfuryl alcohol)/wood veneer prepregs with high shelf lives and their subsequent molding into laminated composites.

## 2. Materials and Methods

### 2.1. Raw Materials and Preliminary Characterization

*Pinus elliottii* veneers with 1 mm thickness, an apparent density of ~0.60 g/cm^3^, moisture content of ~12.00%, and porosity of ~50.00% were acquired from EcoFolhas (São Paulo, Brazil) and utilized in the current study. Furfuryl alcohol (FA), characterized by a density of ~1.14 g/cm^3^ and a dynamic viscosity ranging from 5.0 to 10 cP, was employed as a wood impregnant, while maleic anhydride (98% purity; Sigma Aldrich, St. Louis, MO, USA) was utilized as a hardening agent.

### 2.2. Prepreg Manufacturing

In order to achieve a uniform and even spread in the resin throughout the wood veneers, vacuum infusion was employed as a pre-impregnation method, based on prior research conducted by our group [17,22]. To accomplish this, ten veneers measuring 120 mm × 300 mm × 1 mm were stacked on a smooth surface. Following this, spiroducts were cut and connected to inlet and outlet hoses, and a vacuum bag was placed atop the veneers, creating an injection area measuring 250 mm × 300 mm. The inlet hose was connected to a 2 L beaker containing the furfuryl alcohol (FA), while the outlet hose was connected to a pressure vessel, which acted as a Büchner flask, in line with a vacuum pump. The entire injection area was sealed with tacky tape. Thereafter, the vacuum pump was activated and adjusted to a constant pressure of −90 kPa, resulting in a rectilinear flow.

### 2.3. Polymerization Kinetics

To determine the shelf life of the prepregs (wood veneers impregnated with FA), the polymerization kinetics were investigated using differential scanning calorimetry (DSC). The DSC experiments were conducted with a 10 mg sample in a DSC-50 Shimadzu (Kyoto, Japan), instrument under a nitrogen atmosphere (50 mL/min). The materials were enclosed in aluminum crucibles and subjected to non-isothermal heating from 25 to 220 °C using four different heating rates (5, 10, 15, and 20 °C/min). The kinetic parameters of the reactions were evaluated using “Netzsch Thermokinetics: a Software Modulus for the Kinetic Analysis of Thermal Measurements, Version—3.1.” The activation energy (Ea(T)) values were calculated through integral and differential model-free isoconversional methods. The Flynn–Wall–Ozawa (FWO), Friedman (FR), and Kissinger–Akahira–Sunose (KAS) methods were employed to establish the Ea(T) dependency on the conversion degree, a(T) [23]. All the kinetic equations used to calculate shelf life were based on Vyazovkin et al. [23].

The corresponding kinetic parameters were evaluated using a “Multivariate Nonlinear Regression” program, which uses a hybrid Marquardt–Levenberg approach. The best kinetic model was chosen with the least squares and F-test methods. The kinetics model equations employed by the software were based on Cruz et al. [24]. The tests and calculations were carried out following the indications of the ICTAC Kinetics Committee based on Vyazovkin et al. [23]. The kinetics parameters of the best model found were used to predict the behavior of the DSC curves; the curing behavior in different isotherms; the heat flux in isotherms (100, 120, 140, and 160 °C); and finally, the diagram time, temperature, and transition (the TTT of the prepreg).

### 2.4. Unidirectional Laminate Manufacturing

Two configurations were adopted for the manufacturing of the laminates, stackings of 3 unidirectional veneers and 4 unidirectional veneers, in the dimensions mentioned in the previous section. The pre-impregnated veneers were positioned in a press and wrapped with aluminum foil to facilitate subsequent demolding and to act as a heat distributor. A pressure of ~3.0 bar was applied at a constant temperature of ~100 °C for 24 h until complete curing of the resin was achieved. Afterward, a post-curing at ~120 °C was performed in a laboratory oven (Leo Estufas/Porto Alegre, Porto Alegre, Brazil).

### 2.5. Laminate Characterization

The morphology of the laminates was analyzed tangential to direction via scanning electron microscopy (SEM) using MA10 equipment (Zeiss Evo brand, Oberkochen, Germany) operating at 3 kV. The hygroscopic properties of the prismatic laminates (39 mm × 10 mm, radial × longitudinal) were determined following a modified ASTM D570 methodology based on [22].

Five laminates per group were submerged in distilled water and weighed daily for ten days. Fourier-transform infrared (FTIR) spectroscopy coupled with an attenuated total reflection device (ATR, Blagnac, France) using IRSpirit equipment (SHIMADZU, Kyoto, Japan) was used to verify hydrolysis, a phenomenon that can impair interlaminar adhesion in lignocellulosic materials. An average of 32 scans within a 600–4000 cm^−1^ range at a scan interval of 4 cm^−1^ was reported for each sample’s spectrum. Thermal stability was assessed using one sample per specimen with a mass of ~16 mg with a thermogravimetric analyzer (TG) at a heating rate of 10 °C.min^−1^ from room temperature (approximately 20 °C) to 800 °C, under a nitrogen atmosphere, using TA instruments.

To determine the apparent density (ρ), ten prismatic samples per group (170 mm × 25 mm, radial × longitudinal) were measured using a digital caliper with a 0.01 mm resolution and an analytical scale with a 0.001 g resolution. Compressive tests were conducted on six samples for each laminate configuration using a 5 kN load cell, in accordance with ASTM D6641 [25]. The samples were cut to 140 mm × 12.7 mm (radial × longitudinal) and tested at a speed of 1.3 mm/min until rupture was achieved. Tensile tests were performed on five samples (dimensions: 170 mm × 25 mm, radial × longitudinal) for each laminate at a constant speed of 2 mm/min with a 5 kN load cell and pneumatic grips, in accordance with ASTM D3039 [26].

### 2.6. Statistical Analysis

Prior to conducting hygroscopic and mechanical tests, sample size adequacy was ensured, with a minimum analysis power of 80%, based on values from a previous study [22]. Next, homogeneity of variances and data normality were assessed using the Shapiro–Wilk and Q-Q plot tests, respectively. One-way analyses of variance (ANOVAs) were then performed, and when the null hypothesis was rejected, Tukey tests were used for mean comparisons. For data measured over time, such as water uptake, Mauchly’s sphericity and Scheffe tests were used for evaluation. All statistical analyses were conducted at a significance level of 5%.

## 3. Results and Discussion

### 3.1. Polymerization Kinetics and Shelf Life

**Figure 1:**
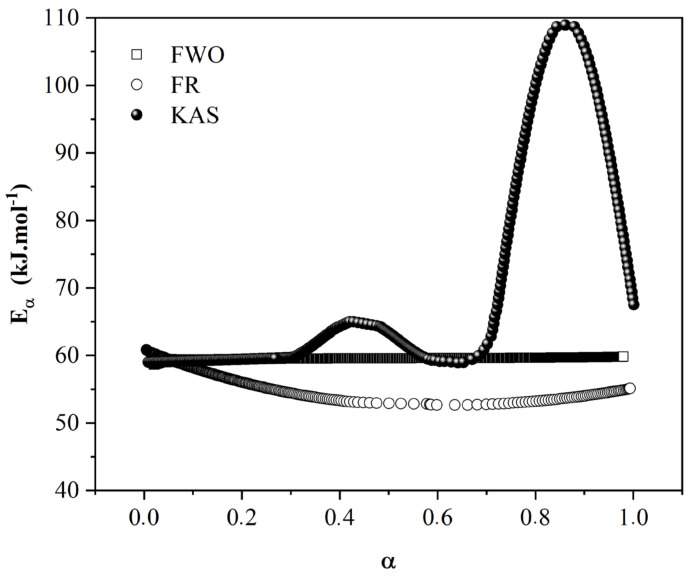
Activation energy (E) versus conversion rate (α) curves for the wood-based prepreg.

Figure 1 shows the activation energy (Eα) versus the conversion rate (α) curves for the novel prepreg. The values were determined using the FWO, FR, and KAS isoconversional model-free methods. Among these methods, KAS better represented the behavior of the system shown in earlier research, with two peaks (65.8 and 109.4 kJ/mol for 0.42 and 0.86 rate conversions, respectively). The FA polymerization mechanism is complex and not limited by the single-step process (as reported before), as suggested by the FR and FWO models [27,28,29].

The relationship between the activation energy and the conversion rate clearly indicates a complex mechanism that involves several chemical steps, with each of them having its own activation energy. As a result, each increasing and decreasing part of the effective activation energy (Eα) can be associated with changes in the rate-limiting steps of the overall polymerization. The obtained Eα values are in agreement with the data previously reported by Guigo et al. [30] at 70 and 102 kJ/mol for 0.46 and 0.85 rate conversions, respectively, which were obtained using the FR method. In this study, the FR and FWO methods did not show conclusive results, with constant activation energy values of 59.2 kJ/mol with the FR method and an Eα decrease of 62.1 to 54.8 kJ/mol (FWO method). This result was to be expected since, according to Vyazovkin et al. [23], compared with the Ozawa–Flynn–Wall method, the Kissinger–Akahira–Sunose method offers a significant improvement in the accuracy of Eα values. Thus, in fact, this kinetic model seems to be more accurate for multi-stage polymerization, similar to what possibly occurs with PFA.

Thus, based on the KAS curve, it is possible to state that the polymerization process follows two consecutive steps. In the initial stage of curing, the Eα is constant at curing degrees of α ≤ 0.30 around 60 kJ/mol, suggesting that a single reaction predominates in this conversion range. According to Guigo et al. [30] and Guo et al. [28], this value indicates that the polycondensation of FA starts with the linear condensation of a hydroxymethyl group (between CH_2_OH groups and C-5 of furan rings) to form methylene linkages, which is similar to the mechanism used to understand the polycondensation of phenol formaldehyde systems [31]. In the continuation of the reaction at α~0.42, there is a sudden increase in Eα o 65.8 kJ/mol, indicating a change in the reaction mechanism during polymerization. According to Falco et al. [29] and Guigo et al. [32], this change can be attributed to the Diels–Alder cycloadditions between oligomers formed previously. Guigo et al. [32] and Guo et al. [28] studied FA polymerization kinetics and found similar Eα values for this step of the reaction (~68 kJ/mol).

There is a decrease in Eα between α = 0.43 and 0.60 from 65.8 to 60.2 kJ/mol, respectively, during polymerization. This decrease is related to the gelation process, in which the growing chains of oligomers, obtained via Diels–Alder cycloadditions, achieve infinite molar mass behavior through connections between furan rings. When polymers gel, a sudden and dramatic increase in viscosity is observed until the molecular weight becomes infinite (degree of polymerization, DP→∞) [24]. This promotes low molecular mobility, which induces a decrease in the overall reaction rate that becomes controlled by the diffusion of small unbranched oligomer chains. This is mainly due to an inherent increase in viscosity after branched cycloadditions and results in a decrease in Eα. The same behavior has been reported before [27,28,29,32].

The reaction exhibits a notable abrupt peak in activation energy (Eα), reaching 109.4 kJ/mol (α = 0.86), followed by a subsequent decrease. Following the gel point, the larger chains that were previously formed via Diels–Alder cycloadditions begin to undergo cycloaddition reactions with smaller chains, leading to the formation of a three-dimensional network and thereby creating a crosslinked structure. During this stage, the mobility of the polymeric macromolecules becomes limited, resulting in an elevation in Eα values for the crosslinking process. The crosslinking, coupled with the consequential restriction of molecular mobility (α > 0.90), manifests as an almost exponential reduction in Eα, which can be attributed to the vitrification of the polymeric chain. According to Guigo et al. [32], vitrification causes a dramatic decrease in molecular mobility, leading to a decrease in the effective activation energy with the increasing extent of the reaction [33]. Based on the results obtained by varying the activation energy as a function of the conversion rate, the non-isothermal kinetic parameters were investigated through multivariate nonlinear regression (Table 1) using the DSC data obtained at different heating rates.

According to Guigo et al. [30] and Guo et al. [28], who obtained similar results to the present study, the FA polymerization reaction follows the autocatalytic mechanism, which corresponds to the formation of the active species for the first stage of condensation. Subsequently, with the reaction’s progress, the diffusional profile is prevalent. In this way, the accuracy of the kinetics models (autocatalytic and diffusional) adopted for determining the kinetic parameters was validated using the F statistical test. Considering the correlation coefficient (r) and F-test values given in Table 1, it can be concluded that the most appropriate models for describing the polymerization mechanism were as follows: Step 1 (Bna—autocatalytic mechanism) and Step 2 (D1—one-dimensional diffusion mechanism). This model presented r and F-test values close to one and activation energy (Eα) values nearest to those found in the KAS models.

**Figure 2:**
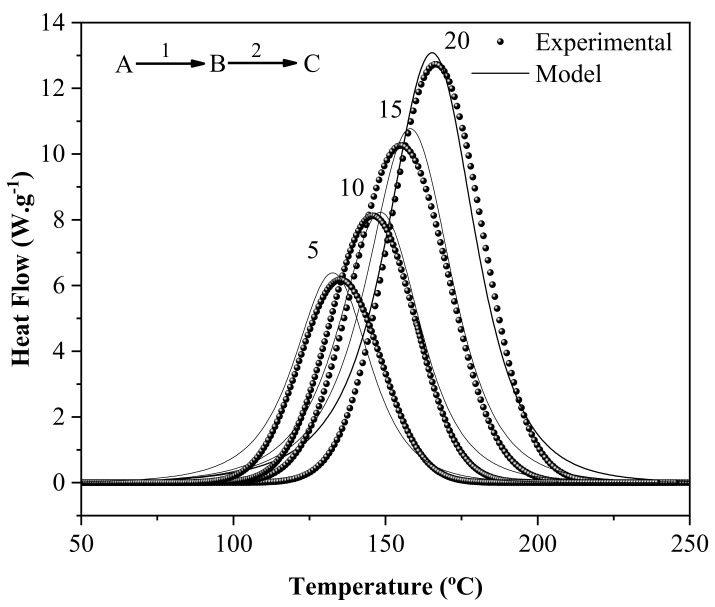
Model prediction (continues line) of prepreg polymerization reactions using multiple-step reactions. First, the autocatalytic mechanism (Prout–Tompkins (Bna) n-th order approach) and, second, diffusional mechanism-based behavior (D1). The different heating rates (in °C/min) employed in the DSC experiments (scatter points) are indicated in each curve.

Figure 2 shows a comparison between results obtained experimentally using DSC and those simulated mathematically with two models in consecutive reactions (autocatalytic and diffusion mechanisms). A good overlap between the experimental and theoretical data was observed, suggesting that the Bna model can satisfactorily describe the polymerization kinetics of the wood-based prepreg developed in this study. The non-isothermal results obtained from the Bna and D1 models in two consecutive reactions (A$\overset{1}{\to }$ B $\overset{2}{\to }$C) were then used as the basis for simulations to predict isothermal polymerization behavior.

**Figure 3:**
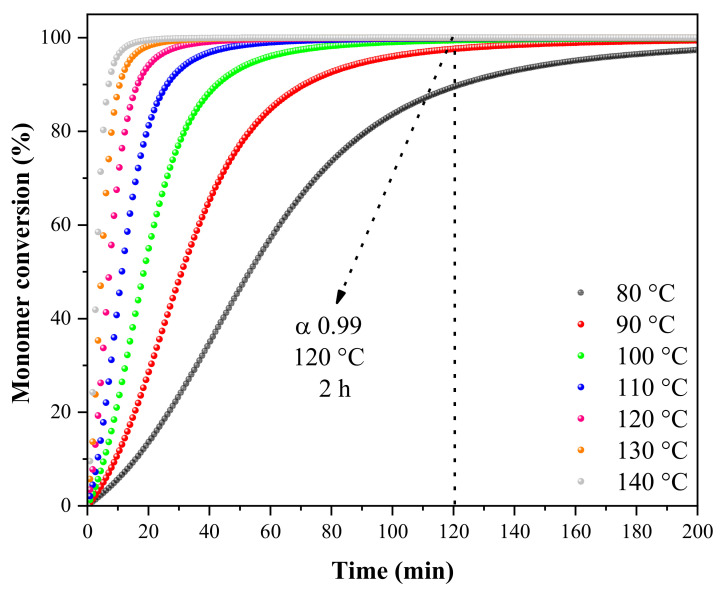
Curing reaction simulation at different temperatures for wood-based prepregs.

Figure 3 shows the effect of temperature on the reactional conversion and, consequently, the time needed to cure the prepreg (achieving high conversion rates at α > 0.98). Based on Figure 3, it can be concluded, for example, that 2 h at a temperature higher than 120 °C is enough to achieve conversion rates of at least 99% of the reaction.

**Figure 4:**
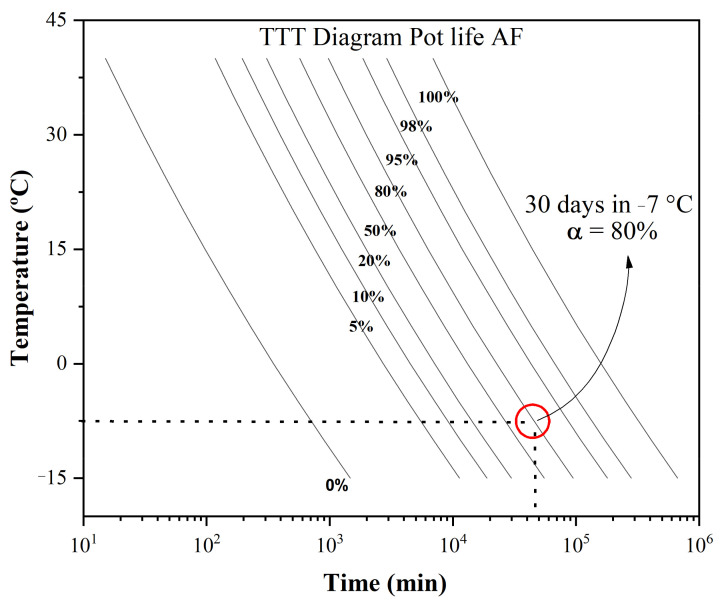
TTT (time–temperature–transition) diagram for prepregs.

With the aim of applying this material as a prepreg—which, after impregnation, can be stacked in a desired way and pressed at certain temperatures to achieve curing and the consolidation of the final laminate—it is of fundamental importance to understand how the advancement of the polymerization reaction rate occurs through the way this prepreg is stored after the slides are impregnated with FA. Therefore, Figure 4 presents a TTT diagram (time, temperature, and transition) obtained using the non-isothermal results, which were then used in simulations to predict the polymerization behavior during prepreg storage at low temperatures (−15 °C to 35 °C). These temperatures simulate a possible prepreg transport/storage condition for later use, delaying wood-based prepreg polymerization.

Several degrees of cure values were used to obtain the relationship between T and t at the fixed degree of cure. Prepreg TTT cure diagrams can provide crucial information regarding reaction rates as functions of time and temperature. For example, after stocking prepreg at a temperature of −7 °C, it would take approximately 30 days to reach a reaction rate of 80% polymerization, as can be seen in detail in Figure 4.

### 3.2. Laminate Morphology and Water Absorption

**Figure 5:**
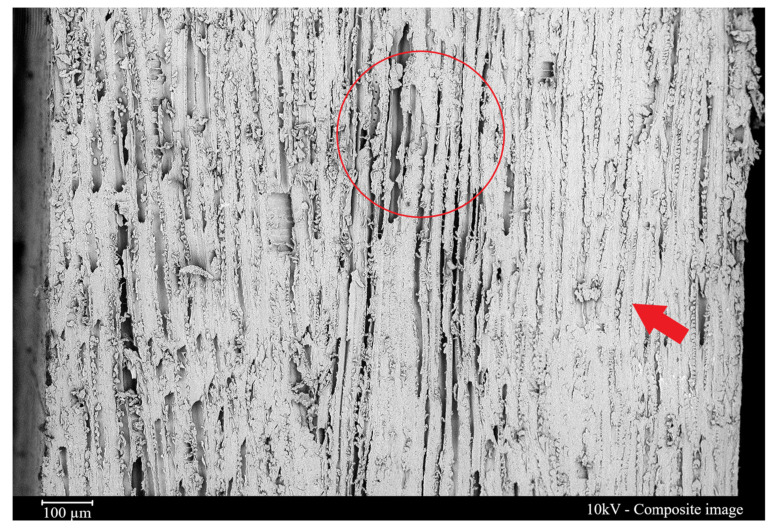
Scanning electron microscopy of the 4-layer wood/poly(furfuryl alcohol) laminate. Where: Arrow represents resin infiltration and circle represents the interface.

Since wood veneers have low permeability compared with traditional materials, some issues during the manufacturing process may occur because of fluid absorption and swelling, which may worsen the properties of the composite. In this sense, the impregnation of wood veneers with poly(furfuryl alcohol) resin through vacuum infusion seems to be a suitable alternative for this purpose. In fact, good impregnation of the poly(furfuryl alcohol) resin was visualized for wood tracheids with SEM images of the four-layer composite (red arrow in Figure 5), verifying that the resin was able to penetrate the porous structure of the wood, improving its bond with the fibers and resulting in a more homogeneous composite.

Despite its low permeability, the resin flow provided very thin laminas (approximately 1.0 mm) and laminates with desirable low matrix content. These characteristics resulted in a good combination for laminate manufacturing, wherein compression molding has been found to be successful in producing high-quality wood-poly(furfuryl alcohol) laminates. This manufacturing process involves heating the material and applying pressure to the mold, allowing the resin to impregnate the wood fibers more effectively. The resulting laminate did not present a clear interface between the stacked laminas. Instead, the homogeneous structure of the materials was achieved with a high-quality manufacturing process.

The SEM images also showed the heterogeneous fiber architecture commonly found in natural fibers (red circle in Figure 5), which hinders resin flow through fiber fabric, leading to the formation of voids filled with entrapped air [34]. Acosta et al. [22], who manufactured wood–jute laminates using the vacuum infusion process, also ascribed the high void content to the presence of jute fabrics. The authors felt that this behavior may be a disadvantage while manufacturing hybrid laminates since the ones produced by the authors presented glass layers that were almost fully filled, while the wood veneers were not filled. Regarding bio-based prepregs, Silva et al. [10] processed sisal/epoxy prepregs and achieved prepreg laminas with 3mm thickness through compression molding, which is a substantially higher value than that of the ones obtained in the present study (~1.0 mm).

**Figure 6:**
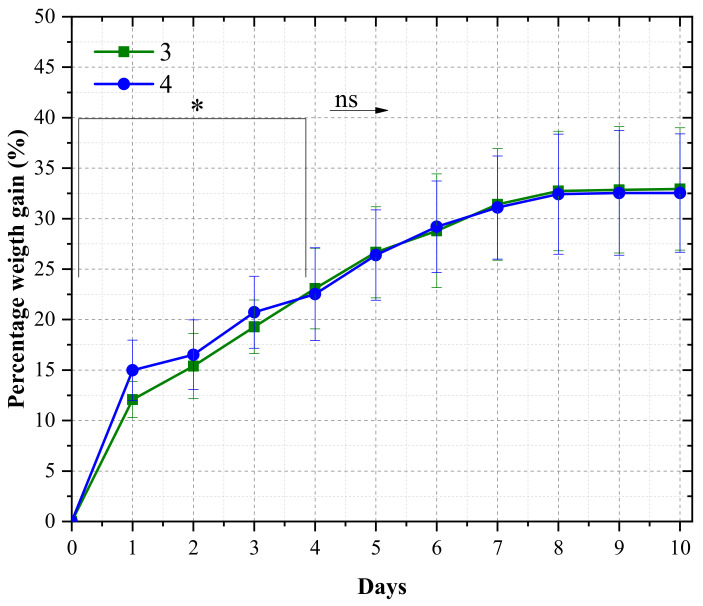
Water uptake of wood/poly(furfuryl alcohol) laminate, wherein (*) represents the days where a statistical difference was observed and ns and arrow represents no statistical difference.

Regarding water absorption, Figure 6 shows the average water percentage uptake for the three- and four-layer laminates over 10 days. High weight gain rates were found for the first day, where 12% and 15% of mass increases were observed in the three- and four-layer composites. In fact, the four-layer laminate showed a higher water uptake for 4 days; after this period, no more statistical differences were observed between the groups. After 8 days, both laminates achieved mass stabilization, with a weight gain of approximately 32% for both groups. The higher water absorption in the first days by the four-layer laminate is probably due to the higher panel thicknesses, wherein the wood surface area was higher than the interlaminar surface area, considering that the lateral ends of the laminates were not sealed with resin after the cutting process. In this sense, recent research corroborates how weight gain can mostly be ascribed to exposed wood, which is the most prone to absorbing water [9,35].

When compared with other wood laminates manufactured with conventional materials, the poly(furfuryl alcohol) matrix was beneficial against water absorption. In fact, in the last study by the group in [22], one, three, and five wood-veneer laminates were produced via the vacuum infusion process using unsaturated isophthalic polyester resin as a matrix, achieving 57, 75, and 88% of weight gain at the end of 10 days. The achieved values in the present study are substantially smaller (≥15%), pointing to a more homogeneous material with fewer voids and lower wettability than the classical non-sustainable composites, making it difficult for the moisture to penetrate the wood veneers because of good adhesion between the reinforcement and the matrix. In fact, the achieved values are much smaller than those reported for structural wood-based laminates, such as LVL [36,37], OSB [38], and plywood [39,40].

### 3.3. Chemical and Thermal Characterization

**Figure 7:**
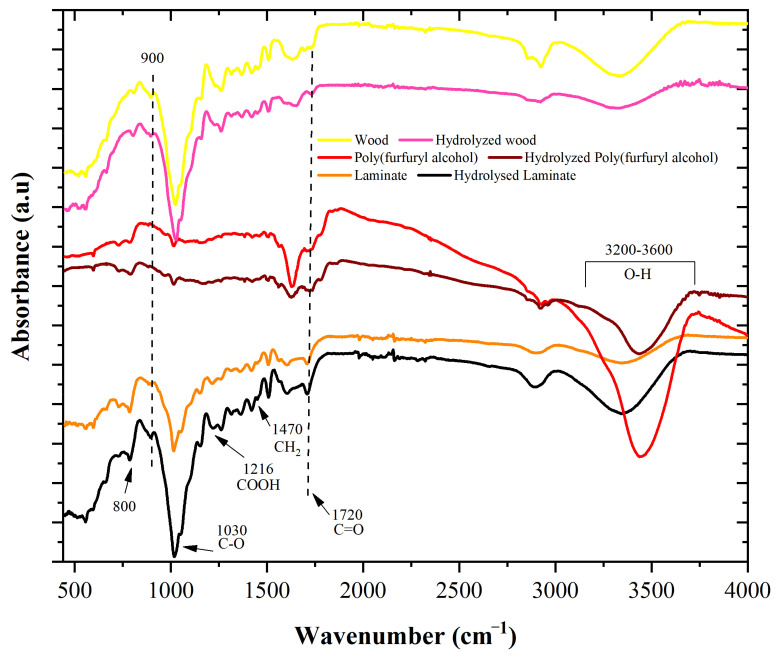
FT-IR spectra of non-hydrolyzed and hydrolyzed wood, poly(furfuryl alcohol), and manufactured laminate.

Figure 7 shows the infrared spectra of the wood, the poly(furfuryl alcohol), and the fabricated laminate. All samples were subject to hydrolysis, and the spectra were analyzed before and after the degradation process. The 1720 cm^−1^ band in the infrared spectrum of wood is typically associated with the stretching vibration of non-conjugated C=O bonds in carbonyl functional groups, such as ketones and aldehyde carbonyl functional groups, which can be present in both hemicellulose and lignin. Hydrolysis in wood involves the breakdown of these polysaccharides into their constituent monosaccharides, and the chemical reactions involved in this process can result in changes in the infrared spectrum of the wood sample. In general, hydrolysis in wood is expected to result in a decrease in the intensity of this band since the carbonyl functional groups are typically reduced or eliminated during the hydrolysis process as a result of the breakdown of these functional groups into smaller molecules or the removal of the functional groups. In fact, the hydrolyzed laminate did not show a change in this band, which points to the conservation of the laminate chemical structure against hydrolysis.

Moreover, the 800 cm^−1^ peak is typically associated with the bending vibration of the C-H bond in cellulose, while the 900 cm^−1^ peak in the spectrum of wood could indicate the presence of aromatic ring deformation vibrations. The peak located at 1030 cm^−1^ is related to classic polysaccharide bonds (C-O), indicating the presence of cellulose, and the 1216 cm^−1^ peak in the spectrum of the laminate could indicate the presence of lignin since the peak is typically associated with the aromatic skeletal vibration of the C-C bond in lignin, which is a complex polymer made up of phenylpropane units. Regarding the 1470 cm^−1^ peak, this band indicates the presence of aromatic ring vibrations from the furan ring in poly(furfuryl).

Finally, the broad absorption bands in the range between 3200 cm^−1^ and 3600 cm^−1^ are related to the classic hydroxyl present in wood materials because of the high number of hydroxyls in cellulose [41]. Again, it can be seen that the band in the laminate did not present a decrease after the induced hydrolysis process, pointing to the good durability of the composite. Conversely, neither the resin nor the wood veneer presented resistance against the hydrolysis process, pointing to the good synergy of the wood veneer and the poly(furfuryl alcohol) in achieving the previous result.

**Figure 8:**
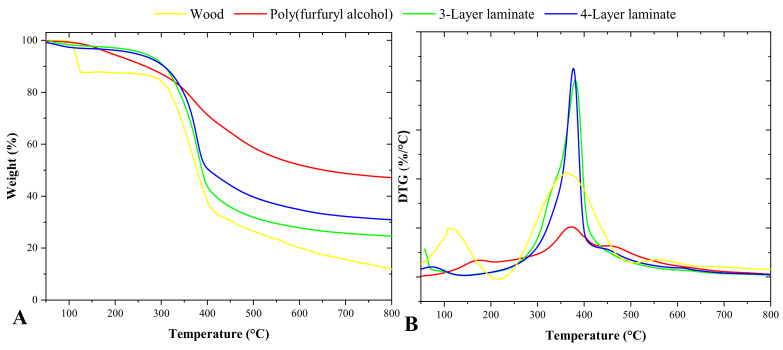
Thermogravimetric analysis (**A**) and DTG (**B**).

Thermogravimetric analysis was conducted to evaluate the influence of poly(furfuryl alcohol) in the degradation of the wood-veneer laminates (Figure 8). The wood samples were degraded in a broad temperature range of 100–800 °C, while a small degradation was observed at approximately 100 °C related to the humidity loss of the material. As the temperature increased, major degradation was initiated at 300 °C, and a peak was observed at 375 °C; in this stage, the fast degradation rate is related to the decomposition of wood components through a series of depolymerization reactions, mainly cellulose and hemicellulose, since lignin can still be degraded at higher temperatures [42].

Table 2 shows thermal events corresponding to various temperatures and their respective mass losses. It can be seen that the mass losses for T_2%_ are quite similar between the different samples, which suggests that these losses are predominantly related to water evaporation. This behavior is to be expected, as the presence of moisture in wood is a common phenomenon, and its thermal release occurs at relatively low temperatures. However, a distinct difference in mass losses becomes evident when considering T_5%_, which represents a higher temperature. At this point, significant material degradation is observed. The mass loss profile, as revealed by thermogravimetric analysis (TGA), shows a marked loss of mass starting around ~200 °C. This is related to the partial degradation of the main wood components, such as cellulose; hemicellulose; and, later, lignin, the latter occurring at higher temperatures. These thermogravimetric events are clearly demonstrated in Table 2, where we can see that the mass-loss onset temperature is lower for raw wood compared with laminates. This observation is an indication of the greater thermal stability of laminates. Laminates, given their manufacturing process, have a denser structure and a polymeric matrix, which makes them more resistant to thermal degradation compared with raw wood. It is also important to note that the residue values after the TGA test were also higher for the laminates compared with the raw wood. This is largely due to the presence of poly(furfuryl alcohol), which has a high residual mass after thermal degradation. The presence of this polymer in the laminate samples contributed to a significant increase in the percentage of residue observed after thermogravimetric analysis.

Regarding poly(furfuryl alcohol) thermal degradation, this exhibited a gradual weight loss as the temperature increased. This weight loss starts at low temperatures because of the evaporation of volatile components, such as water and small organic molecules. As the temperature increases further, furfuryl alcohol begins to decompose and undergo polymerization with the scission of methylene bonds and the formation of compounds such as 2-methyl furan and 2-furfuryl-5-methylfuran. As the temperature increases, furan bonds undergo scission, and volatile compounds form, such as acetone, 2-butanone, and 2-pentanon. As for the wood/poly(furfuryl alcohol), the impregnation of the resin did not alter the decomposition steps of the material; as can be seen in the DTG curves, the peak of degradation of the resin is in the same range of temperature as the main wood components. This behavior led to a similarity between the degradation rates in terms of weight loss in the laminates with wood.

### 3.4. Mechanical Properties

The results of the mechanical tests on the three- and four-layer laminates presented no significant difference in any of the observed properties (Table 3). The densities were 0.937 g/cm^3^ and 0.849 g/cm^3^ for the three- and four-layer laminates, respectively, which were lower than the ones achieved in other studies seeking to create sustainable laminate materials. In this sense, Li et al. [35] achieved densities of 0.961 g/cm^3^ and 0.968 g/cm^3^ using bamboo-bundle-laminated veneer. The mechanical tests conducted on the wood/poly(furfuryl alcohol) prepregs in this study demonstrated good properties when compared with similar research. In this sense, the tensile strength for the three-layer and four-layer composites were 43.8 MPa and 59.6 MPa, respectively. Those values were higher than the ones obtained by Mohammadabadi et al. [6], who manufactured wood-based prepregs for composite laminates using wood strands and thermoplastic resin, obtaining a tensile strength of 32 MPa. However, the same authors also reported a higher tensile modulus (~13.40 GPa) than the ones obtained in this research (7.15 and 7.29 GPa). On the other hand, the reported values for the tensile modulus obtained by Silva et al. [10] with sisal/epoxy prepregs (3.33 GPa) were lower than those achieved for poly(furfuryl alcohol)/wood.

Additionally, the samples also showed good flexural strength and modulus; in fact, the values were similar to those achieved by Fleckenstein et al. [9], who obtained a flexural strength and modulus of approximately 110 MPa and 11.500 MPa, respectively, using phenol formaldehyde resin to manufacture laminated-veneer lumber. However, although the resin used by the authors was also a valuable bio-based material, phenol formaldehyde is known to be carcinogenic.

Regarding compressive strength, wood exhibited greater strength parallel to the fiber direction when subjected to compressive loads. In this sense, poly(furfuryl alcohol) presented an ability to confer a strong bond along the centerline, preventing the delamination of the specimens. Consequently, the laminates manufactured in this study achieved increased compressive strength when compared with other structural laminates.

These results are highly encouraging and have significant implications for the potential use of wood/poly(furfuryl alcohol) prepregs for structural applications where high mechanical strength and durability are required. Furthermore, it is important to note that, in general, the mean strength and stiffness of the fabricated composites were higher than those reported in the literature for structural wood-based laminates such as LVL [43,44].

## 4. Conclusions

The manufacturing of wood/poly(furfuryl alcohol) prepregs via the vacuum infusion process resulted in good impregnation of the veneers, enabling the assembly of laminate composites with properties that are superior to those that were found in previous examples of wood-based composites. Compression molding the prepregs resulted in laminates with homogeneous structures, resulting in low values of water absorption when compared with other structural wood-based laminates (LVL, OSB, and plywood). Furthermore, poly(furfuryl alcohol) and wood presented a good synergy for hydrolysis resistance, and no degradation evidence was seen after the aging test. The composites also presented high compressive, tensile, and flexural properties when compared with other structural wood-based laminates. In conclusion, the good polymerization kinetics, hydrolysis resistance, and mechanical properties of the wood/poly(furfuryl alcohol) prepregs, combined with their ecofriendly attributes, make them an attractive option for manufacturers seeking high-performance, sustainable materials for their products. Future studies will aim to reduce the polymerization capacity of the PFA in order to increase the shelf life of these prepregs, as well as manufacture laminates with a greater number of sheets and evaluate their behavior in fire, biodegradability, and impact tests.

## Figures and Tables

**Table 1:** Kinetics parameters of prepreg obtained using the nonlinear regression method.

Step 1/Step 2	E_1 (T)_ (kJ mol^−1^)	log A_1_ (s^−1^)	E_2 (T)_ (kJ mol^−1^)	log A_2_ (s^−1^)	Correlation Coefficient (r)	F Test
B_na_/D_1_ (n = 1.50, log K_cat_ = 1.25)	55.91	4.04	99.98	1.29	0.97	1.00
B_na_ (n = 1.22, a = 0.58)/D_3_	55.42	5.08	124.28	−0.75	0.98	1.05
C_1B_/D_2_ (n = 1.50, log K_cat_ = 1.26)	55.29	3.95	99.92	0.98	0.98	1.14
C_1B_/D_3_ (n = 1.50, log K_cat_ = 1.27)	55.21	3.94	96.85	0.98	0.98	1.34
C_1B_/D_4_ (n = 1.50, log K_cat_ = 1.27)	55.18	3.93	90.35	−1.44	0.95	2.34
C_nB_/R_2_ (n = 1.50, log K_cat_ = 1.27)	55.18	3.93	90.25	4.94	0.94	4.68
C_nB_/R_3_ (n = 1.49, log K_cat_ = 1.26)	55.22	3.94	102.08	−14.68	0.91	7.23
C_nB_/D_1_ (n = 1.06, log K_cat_ = -4.00)	78.85	7.59	98.25	1.31	0.93	8.35
C_nB_ (n = 1.42, log K_cat_ = 1.29)/D_2_	55.24	3.92	25.21	5.45	0.97	16.63
C_nB_ (n = 1.42, log K_cat_ = 1.29)/D_3_	55.59	4.67	87.89	1.29	0.169	22.83
C_1B_ (log K_cat_ = 0.86)/D_3_	50.51	4.03	99.23	3.26	0.97	29.15
C_1B_ (log K_cat_ = 0.86)/D_2_	54.05	4.01	87.89	2.48	0.96	29.32
B_na_ (n = 1.22, a = 0.58)/D_1_	47.76	3.76	54.22	1.06	0.75	36.32
C_1B_/D_1_ (log K_cat_ = 1.06)	47.76	3.76	41.33	1.06	0.26	45.40
B_na_/D_1_ (n = 1.06, a = 4.00)	47.76	3.76	85.23	1.31	0.23	80.48
B_1_ (n = 1.22, a = 0.58)/D_2_	55.42	5.08	29.78	98.19	0.98	111.75

**Table 2:** Main TG events for the wood, polymers, and laminates.

Group	T_2%_	T_5%_	T_50%_	Residue (%)
Wood	106.60	113.24	366.40	12.60
Poly(furfuryl alcohol)	142.59	190.20	653.56	47.12
3-Layer laminate	118.90	261.88	388.56	24.67
4-Layer laminate	90.57	241.94	403.67	30.97

**Table 3:** Physical and mechanical properties of the laminates. Where: different letters represent statistical difference.

Group	Density (g/cm^3^)	Compressive Strength (MPa)	Tensile Strength (MPa)	Tensile Modulus (MPa)	Flexural Modulus (MPa)	Flexural Strength (MPa)
3	0.937 ^(0.087 a)^	92.5 ^(7.25 a)^	43.8 ^(11.2 a)^	7151.0 ^(656 a)^	11953.00 ^(1021 a)^	108.15 ^(33.4 a)^
4	0.849 ^(0.084 a)^	100.0 ^(5.38 a)^	59.6 ^(11.4 a)^	7297.0 ^(976 a)^	13627.00 ^(843 a)^	105.54 ^(17.60 a)^

## Data Availability

The study did not report any data.
